# Short-term outcomes of preoperative chemotherapy with docetaxel, oxaliplatin, and S-1 for gastric cancer with extensive lymph node metastasis (JCOG1704)

**DOI:** 10.1007/s10120-023-01453-7

**Published:** 2024-01-05

**Authors:** Yukinori Kurokawa, Yuichiro Doki, Ryo Kitabayashi, Takaki Yoshikawa, Takashi Nomura, Kunihiro Tsuji, Masahiro Goto, Haruhiko Cho, Jun Hihara, Naoki Hiki, Souya Nunobe, Junki Mizusawa, Narikazu Boku, Masanori Terashima

**Affiliations:** 1 Department of Gastroenterological Surgery, Osaka University Graduate School of Medicine, 2-2-E2, Yamadaoka, Suita, Osaka 565-0871 Japan; 2 Japan Clinical Oncology Group Data Center/Operations Office, National Cancer Center Hospital, Tokyo, Japan; 3 Department of Gastric Surgery, National Cancer Center Hospital, Tokyo, Japan; 4 Department of Surgery, Yamagata Prefectural Central Hospital, Yamagata, Japan; 5 Department of Medical Oncology, Ishikawa Prefectural Chuo Hospital, Kanazawa, Japan; 6 Cancer Chemotherapy Center, Osaka Medical and Pharmaceutical University Hospital, Osaka, Japan; 7 Department of Surgery, Tokyo Metropolitan Cancer and Infectious Diseases Center Komagome Hospital, Tokyo, Japan; 8 Department of Surgery, Hiroshima City North Medical Center Asa Citizens Hospital, Hiroshima, Japan; 9 Department of Upper Gastrointestinal Surgery, Kitasato University School of Medicine, Sagamihara, Japan; 10 Department of Gastroenterological Surgery, Cancer Institute Hospital, Japanese Foundation for Cancer Research, Tokyo, Japan; 11 Department of Oncology and General Medicine, IMSUT Hospital, Institute of Medical Science, University of Tokyo, Tokyo, Japan; 12 Division of Gastric Surgery, Shizuoka Cancer Center, Nagaizumi, Japan

**Keywords:** Preoperative DOS, Neoadjuvant chemotherapy, Perioperative chemotherapy, Bulky nodal involvement, Para-aortic nodal metastases

## Abstract

**Background:**

The prognosis for marginally resectable gastric cancer with extensive lymph node metastasis (ELM) remains unfavorable, even after R0 resection. To assess the safety and efficacy of preoperative docetaxel, oxaliplatin, and S-1 (DOS), we conducted a multicenter phase II trial.

**Methods:**

Eligibility criteria included histologically proven HER2-negative gastric adenocarcinoma with bulky nodal (bulky N) involvement around major branched arteries or para-aortic node (PAN) metastases. Patients received three cycles of docetaxel (40 mg/m^2^, day 1), oxaliplatin (100 mg/m^2^, day 1), and S-1 (80–120 mg/body, days 1–14), followed by gastrectomy with D2 plus PAN dissection. Subsequently, patients underwent postoperative chemotherapy with S-1 for 1 year. The primary endpoint was major (grade ≥ 2a) pathological response rate (pRR) according to the Japanese Classification of Gastric Carcinoma criteria.

**Results:**

Between October 2018 and March 2022, 47 patients (bulky N, 20; PAN, 17; both, 10) were enrolled in the trial. One patient was ineligible. Another declined any protocol treatments before initiation. Among the 45 eligible patients who initiated DOS chemotherapy, 44 (98%) completed 3 cycles and 42 (93%) underwent R0 resection. Major pRR and pathological complete response rates among the 46 eligible patients, including the patient who declined treatment, were 57% (26/46) and 24% (11/46), respectively. Common grade 3 or 4 toxicities were neutropenia (24%), anorexia (16%), febrile neutropenia (9%), and diarrhea (9%). No treatment-related deaths occurred.

**Conclusions:**

Preoperative chemotherapy with DOS yielded favorable pathological responses with an acceptable toxicity profile. This multimodal approach is highly promising for treating gastric cancer with ELM.

**Supplementary Information:**

The online version contains supplementary material available at 10.1007/s10120-023-01453-7.

## Introduction

Gastric cancer is the fourth leading cause of cancer deaths worldwide [1]. The only curative treatment for gastric cancer is surgical resection [2–4]. Patients with gastric cancer sometimes have para-aortic lymph node metastases or bulky lymph node metastases located along the main arteries such as the celiac, splenic, common, or proper hepatic arteries. The prognosis of patients with gastric cancer who have such extensive lymph node metastasis (ELM) is extremely poor, even after R0 resection. To improve the outcomes of patients with marginally resectable disease, the Stomach Cancer Study Group of the Japan Clinical Oncology Group (SCSG/JCOG) conducted three phase II trials (JCOG0001, JCOG0405, and JCOG1002) of preoperative chemotherapy with or without postoperative chemotherapy after 2000 [5–7]. Based on the results of these three trials, the current Japanese Gastric Cancer Treatment Guidelines state that a preoperative doublet regimen of cisplatin plus S-1 (CS) for two cycles followed by extended surgery and postoperative S-1 for eight cycles is the standard treatment for gastric cancer with ELM [8].

However, in the West, perioperative chemotherapy with fluorouracil, leucovorin, oxaliplatin, and docetaxel (FLOT) is the standard treatment for resectable gastric cancer (except for cT1N0) according to the results of a German phase III trial (FLOT4) [9]. In addition, a preoperative triplet regimen of docetaxel, oxaliplatin, and S-1 (DOS) for three cycles followed by postoperative S-1 for eight cycles resulted in a significant improvement in progression-free survival (PFS) and overall survival (OS) compared with postoperative S-1 for eight cycles after upfront surgery for cT2-3N1 or cT4 gastric cancer in a Korean phase III trial (PRODIGY) [10, 11]. An Osaka University research group modified the dose of docetaxel from 50 to 40 mg/m^2^ due to concerns about neutropenia [12]. They reported sufficient antitumor effects and tolerable safety in a phase II trial of preoperative DOS for cStage III gastric or esophagogastric junction adenocarcinoma [13]. Based on these findings, the SCSG/JCOG initiated a new phase II trial (JCOG1704) to evaluate the safety and efficacy of preoperative DOS for gastric cancer with ELM.

## Patients and methods

### Patients

JCOG1704 was a multicenter phase II trial involving 22 institutions within the SCSG/JCOG. The eligibility and exclusion criteria for this trial were previously reported [14]. Briefly, histologically proven HER2-negative gastric adenocarcinoma with lymph node metastasis (larger than 1 cm based on enhanced computed tomography (CT)) in para-aortic node (PAN) stations (no. 16a2 or 16b1) or bulky lymph node (bulky N) metastasis (one lymph node larger than 3 cm or two lymph nodes larger than 1.5 cm based on enhanced CT) in stations no. 7, 8a, 9, 11, 12a, or 14v was eligible. Conversely, patients with distant metastasis except in lymph node stations no. 16a2 or 16b1, esophageal invasion of more than 3 cm, or remnant gastric cancer were ineligible. Macroscopic (Borrmann) type 4 or large (≥ 8 cm) type 3 tumors were excluded from the eligibility criteria of this trial, because they usually exhibit peritoneal metastases even after R0 resection. Other patient eligibility criteria included: age from 20 to 75 years, ECOG performance status of 0 or 1, sufficient oral intake with or without bypass surgery, and appropriate organ function (neutrophil count ≥ 2000/mm^3^, hemoglobin ≥ 8.0 g/dL, platelet count ≥ 100,000/mm^3^, serum total bilirubin ≤ 1.5 mg/dL, serum aspartate aminotransferase (AST) ≤ 100 IU/L, serum alanine aminotransferase (ALT) ≤ 100 IU/L, and creatinine clearance (CCr) ≥ 50 mL/min). Patients with a history of gastric cancer surgery except for bypass surgery or endoscopic resection or a history of chemotherapy or radiation therapy for other malignancies were excluded.

Staging laparoscopy or laparotomy before enrollment was mandatory to confirm the absence of peritoneal metastasis (P0) and negative cytology in peritoneal lavage fluid (CY0). All patients provided written informed consent. The JCOG Protocol Review Committee and the Certified Review Board of the National Cancer Center Hospital approved this trial protocol, which was registered in the Japan Registry of Clinical Trials (jRCTs031180028).

### Preoperative chemotherapy

Within 2 weeks after enrollment, patients received preoperative chemotherapy consisting of intravenous docetaxel (40 mg/m^2^) and oxaliplatin (100 mg/m^2^) on day 1 and oral S-1 (80 mg/m^2^/day adjusted based on body surface area (< 1.25 m^2^, 80 mg/day; 1.25–1.5 m^2^, 100 mg/day; and > 1.5 m^2^, 120 mg/day) on days 1–14, in a 3-week cycle. This treatment cycle was repeated three times based on the PRODIGY trial, unless clear disease progression or unacceptable toxicity was observed. Tumor response was assessed after cycles 1 and 3 of preoperative chemotherapy using enhanced abdominal CT. The specific criteria for dose reduction and suspension of S-1 administration were previously documented [14].

### Surgery

Within 4 (recommended) to 8 weeks after the last administration of S-1, we performed surgery on patients who were amenable to R0 resection based on enhanced abdominal and chest CT findings and had satisfactory organ function as indicated by blood tests conducted within 2 weeks before surgery. In this trial, it was mandatory for each patient to undergo either distal or total gastrectomy with D2 dissection in addition to the complete PAN dissection of stations no. 16a2 and 16b1, as previously described [15]. PAN dissection. The spleen was preserved unless the tumor involved the greater curvature of the upper stomach or there was suspected metastasis in the splenic hilar node (no. 10). Any neighboring organ(s) invaded by the tumor were removed to ensure R0 resection. The method of reconstruction was not prespecified. Laparoscopic gastrectomy and complete bursectomy were not permitted. If laparotomy revealed unresectable metastases, including positive cytology in peritoneal lavage fluid (CY1), the protocol treatment was terminated at that point.

### Postoperative chemotherapy

Within 6 weeks after achieving R0 resection, patients began receiving postoperative chemotherapy consisting of S-1 (80 mg/m^2^/day) on days 1–28, repeated every 6 weeks until 1 year after surgery, unless the pathological response to preoperative DOS chemotherapy was graded as 0 (no evident treatment effect) on the basis of the Japanese Classification of Gastric Carcinoma (JCGC) criteria. If postoperative S-1 administration could not start within 12 weeks after surgery due to any reason, the protocol treatment was terminated at that point.

Patients were subject to a fixed follow-up schedule for at least 5 years after surgery. Blood tests measuring CEA and CA19-9 were repeated every 3 months during the initial 3 years and then every 6 months for the subsequent 2 years. Enhanced abdominal CT was performed every 6 months for the first 3 years and annually for the following 2 years. Chest X-ray and upper esophagogastroduodenoscopy (except after total gastrectomy) were carried out annually.

### Outcomes

Pathological response was assessed based on both the JCGC and Becker regression criteria [16, 17]. In brief, JCGC grade 3 indicates the absence of viable tumor cells, grade 2b indicates viable tumor cells in less than 1/10 of the tumor area, grade 2a indicates viable tumor cells in 1/10 to 1/3 of the tumor area, and grade 1b indicates viable tumor cells in 1/3 to 2/3 of the tumor area. According to the Becker regression criteria of tumor regression grade (TRG), grade 1a indicates the absence of residual tumor cells, grade 1b indicates less than 10% residual tumor cells, and grade 2 indicates 10–50% residual tumor cells.

The primary endpoint for this trial was the major pathological response rate (pRR), defined as JCGC grade 3, 2b, or 2a. The secondary endpoints included OS, relapse-free survival for patients undergoing R0 resection, proportion of patients with R0 resection, preoperative chemotherapy response rate according to RECIST, version 1.0, proportion of patients completing both preoperative chemotherapy and protocol-specified surgery, proportion of patients completing all protocol-directed treatments, and adverse events. Adverse events were assessed based on the Common Terminology Criteria for Adverse Events (CTCAE), version 4.0. The Clavien–Dindo classification was used to evaluate postoperative morbidities [18, 19]. Clinical and pathological staging was evaluated according to the 15th edition of the JCGC.

### Statistical analysis

In the previous JCOG0405 trial with the identical patient eligibility criteria, major pRR was 27%. Employing a SWOG two-stage study design [20], with 80% statistical power for an anticipated major pRR of 40% with a threshold of 25% and one-sided alpha of 10%, the projected sample size was determined to be 45. Therefore, we planned to enroll 50 patients, accounting for 5 potentially ineligible participants. If 5 or more patients among the initial 25 patients had a major pathological response during the first stage, an additional 25 patients would be enrolled for the second stage. At the second-stage analysis for all patients, preoperative DOS chemotherapy would be considered a promising regimen if the preset threshold value of 25% was rejected with a one-sided alpha of 10%. In other words, if 17 or more of 50 patients had a major pathological response, preoperative DOS chemotherapy would be considered a promising regimen. The confidence interval for pRR was estimated using Clopper-Pearson method. All analyses were conducted based on the intention-to-treat principle using SAS^®^ version 9.4 or higher (SAS Institute, Cary, NC, USA).

## Results

**Fig. 1:**
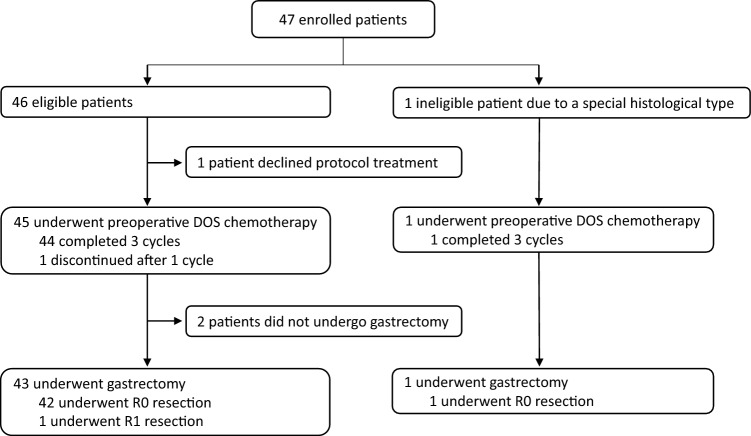
Study flowchart

Between December 2018 and March 2022, a total of 47 patients (20 with bulky N, 17 with PAN, and 10 with both) were enrolled in this trial (Fig. 1). Among these patients, one was ineligible due to a special histological type (adenocarcinoma with enteroblastic differentiation). The baseline characteristics of the 47 patients are shown in Table 1. However, one eligible patient declined any protocol treatments before initiation. Among the 45 eligible patients who initiated DOS chemotherapy, 44 patients (98%) completed 3 cycles, whereas 1 patient discontinued after 1 cycle due to a grade 3 thromboembolic event. The relative dose intensity was 94.9% for docetaxel, 94.2% for oxaliplatin, and 87.7% for S-1. Adverse events experienced during preoperative DOS chemotherapy among the 45 eligible patients who initiated DOS are detailed in Table 2. Notably, grade 3 or 4 toxicities attributed to DOS included neutropenia in 11 patients (24%), anorexia in 7 (16%), febrile neutropenia in 4 (9%), and diarrhea in 4 (9%). There were no treatment-related deaths.

**Table 1:** Baseline characteristics of the study patients

	(n = 47)
Age, years	
Median (range)	67 (20–74)
Sex	
Male	39 (83%)
Female	8 (17%)
Macroscopic type	
0	5 (11%)
1	1 (2%)
2	24 (51%)
3	16 (34%)
5	1 (2%)
Histological type	
Differentiated	33 (70%)
Undifferentiated	14 (30%)
Location of tumor	
Upper third	14 (30%)
Middle third	16 (34%)
Lower third	17 (36%)
Esophageal involvement	
Absent	43 (91%)
Present	4 (9%)
cT category	
T1b	2 (4%)
T2	3 (6%)
T3	9 (19%)
T4a	31 (66%)
T4b	2 (4%)
cN category	
N1	7 (15%)
N2	28 (60%)
N3	12 (26%)
cM category	
M0	20 (43%)
M1	27 (57%)
cStage	
III	19 (40%)
IVA	1 (2%)
IVB	27 (57%)
Node status	
Bulky N only	20 (43%)
PAN only	17 (36%)
Both bulky N and PAN	10 (21%)

N, node; PAN, para-aortic node

**Table 2:** Common adverse events during preoperative chemotherapy

Term	Grade 0	Grade 1	Grade 2	Grade 3	Grade 4	% Grade 3–4
Neutrophile count decreased	14	10	10	6	5	24.4
Anemia	19	22	4	0	0	0
Platelet count decreased	35	8	2	0	0	0
Blood bilirubin increased	42	3	0	0	0	0
AST increased	16	28	1	0	0	0
ALT increased	26	17	2	0	0	0
Febrile neutropenia	41	–	–	4	0	8.9
Fatigue	29	8	6	2	–	4.4
Anorexia	12	15	11	7	0	15.6
Diarrhea	28	7	6	4	0	8.9
Mucositis oral	36	4	3	2	0	4.4
Nausea	28	10	6	1	–	2.2
Vomiting	40	3	2	0	0	0
Peripheral neuropathy	32	11	0	2	0	4.4
Upper respiratory infection	44	–	0	1	0	2.2
Dysgeusia	34	7	4	–	–	–
Alopecia	27	10	8	–	–	–

AST, aspartate aminotransferase; ALT, alanine aminotransferase

Among the 45 eligible patients who initiated DOS chemotherapy, 43 (96%) underwent surgery after chemotherapy and 42 (93%) underwent R0 resection. One patient underwent R1 resection due to positive cytology (CY1). Two patients were not amenable to R0 resection on the basis of a preoperative assessment. The pathological findings in the resected specimens are summarized in Table 3. Postoperative complications (Clavien–Dindo grade III or higher) were pancreatic fistula in two patients (5%), abdominal abscess in one (2%), anastomotic leakage in one (2%), and pleural effusion in one (2%). There were no in-hospital deaths.

**Table 3:** Pathological findings in the resected specimens

	(n = 44)
ypT category	
T0	11 (25%)
T1	11 (25%)
T2	3 (7%)
T3	13 (30%)
T4a	5 (11%)
T4b	1 (2%)
ypN category	
N0	19 (43%)
N1	11 (25%)
N2	7 (16%)
N3	7 (16%)
M category	
M0	42 (96%)
M1	2 (4%)
ypStage	
Unclassified (T0 N0)	11 (25%)
IA	8 (18%)
IB	1 (2%)
IIA	5 (11%)
IIB	6 (14%)
IIIA	5 (11%)
IIIB	4 (9%)
IIIC	1 (2%)
IV	3 (7%)
Residual tumor	
R0	43 (98%)
R1	1 (2%)

Responses to preoperative chemotherapy based on RECIST, version 1.0 were partial response (PR) in 30 patients, stable disease (SD) in 12, and progressive disease (PD) in 2, resulting in a response rate of 65% (30/46) among all eligible patients. Pathological responses of the primary tumor among the 43 eligible patients who underwent surgical resection are outlined in Table 4. Briefly, major (JCGC grade ≥ 2a) pRR and pathological complete response (pCR) were attained in 26 (60%) and 11 (26%) patients, respectively. Regarding JCGC grade 3 cases, there was a trend toward a higher prevalence (45%) of cases located in the upper third of the stomach (Supplementary Table S1). Concerning JCGC grade 1a cases, we observed a trend toward a higher proportion (69%) of cases with bulky N only and a lower proportion (15%) of cases with PAN only (Supplementary Table S2). Among 46 eligible patients, including 1 patient who declined any protocol treatments, major pRR was 57% (80% CI, 46–67%; 95% CI, 41.1–71.1%), with a statistical significance against the threshold of 25% (*p* < 0.0001). Patients with differentiated histological types had a major pRR of 61% (20/33), while patients with undifferentiated types had a rate of 46% (6/13) (*p* = 0.51). Furthermore, neither tumor location nor node status (bulky N, PAN, or both) significantly affected major pRR (*p* = 0.43 and *p* = 0.43, respectively). When evaluating pathological responses according to the Becker criteria, major (TRG 1a or 1b) and minor (TRG 1a, 1b, or 2) pRR were 39% (18/46) and 65% (30/46), respectively. Pathological responses across JCOG phase II trials targeting gastric cancer with ELM are summarized in Table 5.

**Table 4:** Pathological responses of the primary tumor

	Grade 0	Grade 1a	Grade 1b	Grade 2a	Grade 2b	Grade 3
Overall (n = 43)	0	13	4	8	7	11
Differentiated type (n = 31)	0	9	2	6	7	7
Undifferentiated type (n = 12)	0	4	2	2	0	4
Upper third (n = 14)	0	4	0	1	4	5
Middle third (n = 16)	0	5	3	4	1	3
Lower third (n = 13)	0	4	1	3	2	3
Bulky N only (n = 19)	0	9	0	4	1	5
PAN only (n = 15)	0	2	4	2	3	4
Both bulky N and PAN (n = 9)	0	2	0	2	3	2

Pathological response was assessed according to the Japanese Classification of Gastric Carcinoma criteria

N, node; PAN, para-aortic node

**Table 5:** Summary of pathological responses across JCOG phase II trials

Regimen of preoperative chemotherapy	Minor pRR (Grade ≥ 1b)	Major pRR (Grade ≥ 2a)	pCR rate (Grade 3)
Irinotecan + cisplatin (JCOG0001)	15% (8/55)	11% (6/55)	2% (1/55)
Cisplatin + S-1 (JCOG0405)	51% (26/51)	27% (14/51)	2% (1/51)
Docetaxel + cisplatin + S-1 (JCOG1002)	50% (26/52)	35% (18/52)	2% (1/52)
Docetaxel + oxaliplatin + S-1 (JCOG1704)	65% (30/46)	57% (26/46)	24% (11/46)

Pathological response was assessed according to the Japanese Classification of Gastric Carcinoma criteria

pRR, pathological response rate; pCR, pathological complete response

## Discussion

In this multicenter phase II trial, preoperative DOS chemotherapy for marginally resectable gastric cancer with ELM exhibited much greater efficacy than initially anticipated. Along with the proportion of patients with R0 resection exceeding 90% within this patient population with highly advanced disease, the substantial pathological impact of DOS chemotherapy, with major pRR of 57% and pCR rate of 24%, was particularly notable. The chemotherapy regimen could be safely managed while maintaining high relative dose intensities for all three drugs. Importantly, no treatment-related deaths were observed during preoperative chemotherapy or after extended surgery involving PAN dissection. This outcome provides robust support for the use of this multimodal treatment approach for this category of gastric cancer, even in the absence of subsequent confirmation via phase III trials. In addition, it is worth noting that the DOS regimen could potentially expand the eligibility for conversion surgery in cStage IV patients with other types of distant metastasis, leading to improved long-term outcomes.

In the context of marginally resectable gastric cancer with ELM, JCOG has consistently conducted phase II trials to find the most promising preoperative chemotherapy regimens while adhering to the same eligibility criteria. In the initial JCOG0001 trial, preoperative chemotherapy consisting of irinotecan plus cisplatin was hampered by significant toxicity, evidenced by a mortality rate of 5.5% [5]. Nonetheless, minor (JCGC grade ≥ 1b) pRR was only 15%. The subsequent JCOG0405 trial achieved the establishment of a tentative standard treatment for this category of gastric cancer. Preoperative chemotherapy with CS yielded higher pRRs (minor, 51%; major, 27%) and a 3-year OS rate of 59% without any mortality [6]. However, the subsequent JCOG1002 trial of a triplet regimen consisting of docetaxel plus CS (DCS) did not yield better results than JCOG0405 [7]. The failure of DCS might be attributed to the lower dose intensity of S-1 (280 mg/m^2^/week) and docetaxel (10 mg/m^2^/week). By contrast, the DOS regimen in this JCOG1704 trial incorporated higher dose intensities of S-1 (373 mg/m^2^/week) and docetaxel (13 mg/m^2^/week) than in JCOG1002. The substitution of oxaliplatin for cisplatin might have contributed to increased efficacy [21]. Notably, the DOS triplet regimen yielded major pRR at least 30% higher and pCR rate at least 20% higher than CS in JCOG0405.

In the West, FLOT has been the standard perioperative chemotherapy regimen for resectable gastric cancer [9]. In the phase II FLOT4 trial, preoperative FLOT chemotherapy yielded major (TRG 1a or 1b) and minor (TRG 1a, 1b, or 2) pRRs of 32% (47/148) and 47% (70/148), respectively, with pCR rate of 14% (20/148) [22]. These pathological response rates for FLOT were comparatively lower than those for DOS in JCOG1704, even though FLOT4 involved patients with earlier-stage gastric cancer. We believe that the robust efficacy of DOS is potentially attributed to the effect of S-1, considering the lower dose intensity of docetaxel and oxaliplatin in DOS compared with that in FLOT. The recent Korean phase III PRODIGY trial demonstrated that preoperative DOS improves both PFS and OS in cStage II–III gastric cancer compared with postoperative S-1 alone [10, 11]. The antitumor activity of S-1 in contrast to 5-fluorouracil in the FLOT regimen might explain the superior pathological efficacy [23]. In this JCOG1704 trial, the dose of docetaxel on day 1 (40 mg/m^2^) was slightly lower than that (50 mg/m^2^) in the PRODIGY trial, on the basis of a prior Japanese phase II trial [13]. This mild dose reduction might have contributed to the favorable compliance rate without compromising efficacy in this trial. However, it is worth noting the relatively elevated incidences of grade 3 or 4 neutropenia, anorexia, diarrhea, and febrile neutropenia. Thus, careful management of adverse events during DOS chemotherapy is necessary.

The primary endpoint of the JCOG1704 trial was major pRR, which diverged from the primary endpoints of previous JCOG trials targeting the same patient population. When recent analyses were conducted using data from previous JCOG trials of preoperative chemotherapy for marginally resectable gastric cancer, pRR was a superior surrogate indicator for survival when compared to response rate based on RECIST [24]. Furthermore, major (JCGC grade ≥ 2a) pRR was found to be more predictive than the minor (JCGC grade ≥ 1b) pRR for non-type 4 gastric cancer, while the predictive difference between the cutoff of residual tumor < 10% and < 33% was quite small [25]. Recent phase II trials evaluating preoperative treatment in the West have often employed pCR rate as the primary endpoint [26]. In this context, the notably high pCR rate (24%) achieved with DOS in JCOG1704 supports the efficacy of this treatment.

While acknowledging the limitations of a single-arm phase II trial, it is important to note that this category of gastric cancer with ELM is relatively uncommon. Conducting a phase III trial with a large sample size might be impractical given the rarity of this condition. Thus, the current Japanese Gastric Cancer Treatment Guidelines describe preoperative CS followed by extended surgery and postoperative S-1 as the standard treatment for gastric cancer with ELM, on the basis of the findings from the JCOG0405 phase II trial. We believe DOS should replace CS as the new standard regimen for preoperative chemotherapy in gastric cancer with ELM.

## Conclusions

Preoperative DOS chemotherapy for marginally resectable gastric cancer with ELM was associated with an impressive pathological response and a notable proportion of patients who achieved R0 resection. The manageable toxicity profile of preoperative DOS, along with favorable compliance for all three drugs, is noteworthy. In addition, the postoperative morbidity associated with D2 plus PAN dissection following DOS chemotherapy was limited. These findings suggest that this multimodal treatment approach is highly promising for treating gastric cancer with ELM.

### Supplementary Information

Below is the link to the electronic supplementary material.

Supplementary file 1 (DOCX 26 KB)
