# Supplementary material for: Short-term outcomes of preoperative chemotherapy with docetaxel, oxaliplatin, and S-1 for gastric cancer with extensive lymph node metastasis (JCOG1704)
Source: Gastric Cancer. 2024 Jan 5;27(2):366–74. doi: 10.1007/s10120-023-01453-7 (PMC10896774; doi:10.1007/s10120-023-01453-7)
Supplement: Supplementary file 1 — (DOCX 26 KB) [file 10120_2023_1453_MOESM1_ESM.docx]

**Table S1. Details of 11 cases with pathological complete response (JCGC grade 3)**

| Macroscopic type | Histological type | Location of tumor | E-I | cT | cN | Node status | No. of cycles of DOS |
| --- | --- | --- | --- | --- | --- | --- | --- |
| 2 | Undifferentiated | U | + | T3 | N2 | Bulky N only | 3 |
| 2 | Differentiated | L | - | T4a | N1 | PAN only | 3 |
| 2 | Differentiated | U | - | T3 | N2 | Bulky N only | 3 |
| 3 | Undifferentiated | M | - | T4a | N2 | Bulky N only | 3 |
| 3 | Differentiated | M | - | T4a | N1 | Both bulky N and PAN | 3 |
| 2 | Differentiated | U | - | T4a | N2 | PAN only | 3 |
| 0 | Undifferentiated | M | - | T1b | N1 | PAN only | 1 |
| 2 | Differentiated | U | - | T3 | N2 | Both bulky N and PAN | 3 |
| 2 | Undifferentiated | U | - | T4a | N2 | Bulky N only | 3 |
| 2 | Differentiated | L | - | T4a | N1 | PAN only | 3 |
| 3 | Differentiated | L | - | T4a | N3a | Bulky N only | 3 |

JCGC, Japanese Classification of Gastric Carcinoma; U, upper third; M, middle third; L, lower third; E-I, esophageal involvement; PAN, para-aortic node; DOS, docetaxel plus oxaliplatin plus S-1

**Table S2. Details of 13 cases with poor pathological response (JCGC grade 1a)**

| Macroscopic type | Histological type | Location of tumor | E-I | cT | cN | Node status | No. of cycles of DOS |
| --- | --- | --- | --- | --- | --- | --- | --- |
| 2 | Differentiated | L | - | T4a | N3a | Bulky N only | 3 |
| 2 | Undifferentiated | U | - | T4a | N2 | Bulky N only | 3 |
| 2 | Differentiated | M | - | T2 | N3a | PAN only | 3 |
| 3 | Differentiated | U | + | T4a | N2 | Bulky N only | 3 |
| 5 | Differentiated | U | - | T4a | N2 | Bulky N only | 3 |
| 2 | Differentiated | M | - | T3 | N2 | Bulky N only | 3 |
| 3 | Differentiated | L | - | T4a | N2 | Bulky N only | 3 |
| 1 | Differentiated | M | - | T4a | N3a | Both bulky N and PAN | 3 |
| 3 | Undifferentiated | M | - | T4a | N3a | Both bulky N and PAN | 3 |
| 2 | Differentiated | M | - | T4a | N2 | Bulky N only | 3 |
| 2 | Undifferentiated | L | - | T4a | N3a | PAN only | 3 |
| 2 | Undifferentiated | L | - | T4a | N2 | Bulky N only | 3 |
| 3 | Differentiated | U | - | T3 | N2 | Bulky N only | 3 |

JCGC, Japanese Classification of Gastric Carcinoma; U, upper third; M, middle third; L, lower third; E-I, esophageal involvement; PAN, para-aortic node; DOS, docetaxel plus oxaliplatin plus S-1
